# Real-world effectiveness of a new powered stapling system with gripping surface technology on the intraoperative clinical and economic outcomes of gastrectomy for gastric cancer

**DOI:** 10.1186/s12962-024-00534-3

**Published:** 2024-05-06

**Authors:** Honghai Guo, Tao Zheng, Yecheng Lin, Tiange Tang, Zhidong Zhang, Dong Wang, Xuefeng Zhao, Yu Liu, Bibo Tan, Peigang Yang, Yuan Tian, Yong Li, Qun Zhao

**Affiliations:** 1 Third Department of General Surgery, The Fourth Hospital of Hebei Medical University, Shijiazhuang, 050011 Hebei People’s Republic of China; 2 Department of Global Health, School of Public Health, New York University, New York, USA

**Keywords:** Manual staplers, Powered staplers, Gripping surface technology, Gastrectomy, Gastric cancer

## Abstract

**Background:**

Surgical staplers have been widely used to facilitate surgeries, and this study aimed to examine the real-world effectiveness of a new powered stapling system with Gripping Surface Technology (GST) on intraoperative outcomes of gastrectomy for gastric cancer.

**Method:**

The data were extracted from the Fourth Hospital of Hebei Medical University’s (FHHMU) medical records system. Participants (N = 121 patients) were classified into the GST (n = 59) or non-GST group (n = 62), based on the use of the GST system. The intraoperative outcomes such as bleeding were assessed by reviewing video records. T-tests, Chi-square tests, and Mann–Whitney-U tests were used to compare the baseline characteristics between groups. Multivariate logistic regression was conducted for adjusting outcomes to study the effect of variables.

**Results:**

Compared with the non-GST group, the GST group had significantly lower risks for intraoperative bleeding, intraoperative anastomosis intervention rate, intraoperative suture, and intraoperative pression (aORs: 0.0853 (p < 0.0001), 0.076 (p = 0.0003), 0.167 (p = 0.0012), and 0.221 (p = 0.0107), respectively). The GST group also consumed one fewer cartridge than the non-GST group (GST:5 vs non-GST: 6, p = 0.0241).

**Conclusion:**

The use of the GST system was associated with better intraoperative outcomes and lower cartridge consumption in Chinese real-world settings.

## Introduction

Gastric cancer (GC) is ranked the fifth most prevalent cancer and the third most in mortality rate worldwide [1]. China has the highest incidence of GC in the world. It was reported that 679,100 GC cases were newly diagnosed in China in 2015 [2]. The economic burden of gastric cancer on Chinese patients could be catastrophic as the average GC treatment cost is estimated to be US$9,899 between 2012 and 2014 [3].

For patients with GC, surgical resection with adequate lymphadenectomy is the only potentially curative treatment method [4] and laparoscopic gastrectomy is the minimally invasive surgery method [5]. Resecting most of the gastric tissue, gastrectomy includes the pyloric antrum and anastomose with the remaining part of the stomach with the duodenum or jejunum. Complications are one of the biggest concerns to patients during and after an operation. There are immediate consequences during the surgery if proper interventions are not promptly taken [6] while some complications may have adverse postoperative effects. For example, anastomotic bleeding could be a lethal complication for patients who undergo gastrectomy for gastric carcinoma [7] while anastomotic leakage is associated with higher postoperative mortality rates and lower long-time survival [8].

Complications such as bleeding could be caused by malformation or the inappropriate size of staples. An ideal surgical stapling process needs tissues to be fixated and free of slippage, thus the tissues could be lifted and be penetrated properly [9]. However, it could be quite challenging for surgeons to have such desired stapling process in thick tissues. The most difficult component is to identify the thickness of the targeted tissue as the tissue’s “fluidity” would react to the compressive force, which may make it arduous to measure the actual thickness of the targeted tissue [9]. Surgical stapling devices have been widely used in gastrectomy for facilitating tissue approximation and transection [10]. Additionally, stapler devices that require less force from surgeons and provide a consistent compressive force to the tissue would also make surgeries much easier for surgeons [11, 12] therefore, it is always of crucial importance to choose a proper stapler that could assist surgeons during surgeries. The gripping surface technology (GST) system, a new generation stapling system that is composed of the stapler that is powered by the GST system and GST cartridges, provides a superior tissue grip on each reload without causing extra trauma during firing [13]. In this study, we evaluated the economic and clinical effectiveness of the GST system in Chinese hospital settings.

## Methods

### Study design

This study was a retrospective cohort study to compare the outcomes in patients who underwent their first laparoscopic gastrectomy using the GST system versus using a non-GST system in a Chinese tertiary hospital. The study protocol was reviewed and approved by the Ethics Review Board of the Fourth Hospital of Hebei Medical University (FHHMU), which provided the de-identified data to create the study cohort and conduct data analysis.

### Data source

The demographic profile, clinical characteristics, intraoperative outcomes, and laboratory examination results were extracted from the FHHMU’s electronic medical records system. The intraoperative outcomes were assessed by reviewing the video records from the surgical video recording system by trained experienced technicians. In addition, all the video records were independently reviewed twice by different technicians for the accuracy of intraoperative outcomes. Physicians at the study center who were responsible for data collection were trained to monitor and process the documented data to assure all the required data were accurately documented and properly uploaded to the electronic data capture (EDC) system.

### Study cohort

**Fig. 1:**
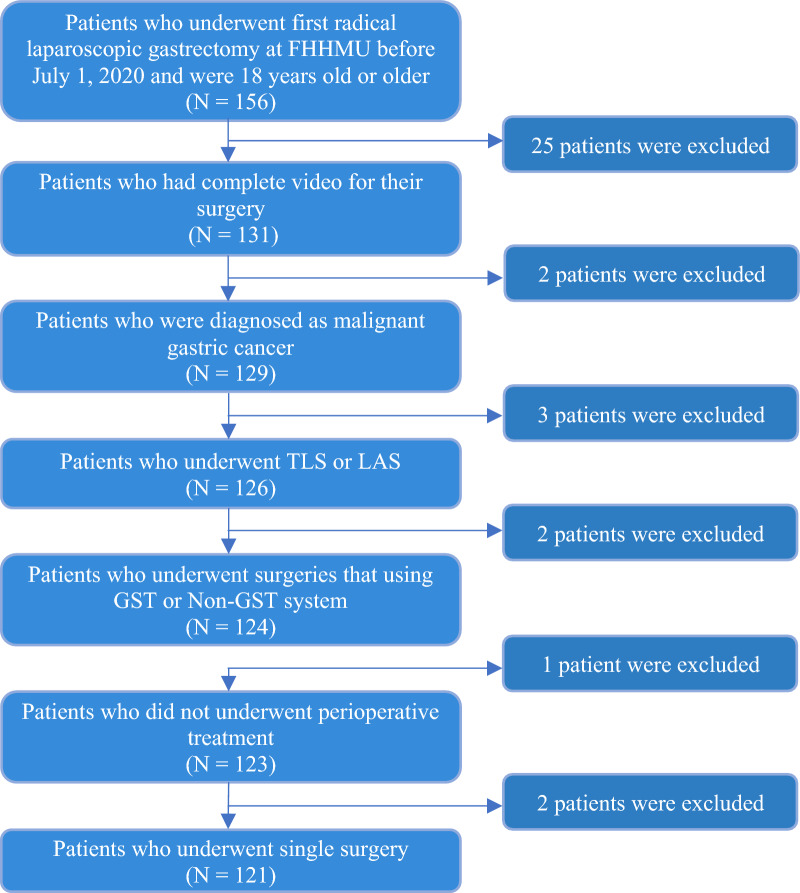
Patient selection flowchart

Eligible participants were defined as patients with the diagnosis of malignant gastric carcinoma who had been admitted to FHHMU between March 2018 and September 2020 and underwent their first laparoscopic gastrectomy using either the GST system or using non-GST system (Fig. 1). The GST system was defined as Echelon Flex powered plus articulating endoscopic linear cutter and Endopath Echelon endoscopic linear cutter reloads with gripping surface technology. The non-GST system was defined as stapling systems without gripping surface technology, including manual (brand name: Panther Healthcare) or powered staplers (Powered Echelon Flex) and their stapler cartridge. The eligible patients were also required to be at least 18 years old at hospital admission. The eligible surgical methods included total laparoscopic surgery (TLS) or laparoscopic-assisted surgery (LAS). The eligible gastrointestinal reconstruction method included gastroduodenostomy (Billroth I), loop gastrojejunostomy (Billroth II) together with Braun enterostomy, and Roux-en-Y esophagojejunostomy. The index date was defined as the date of the surgical operation. The observation period for each patient was up to 30 days after the index date to determine if there was readmission. Patients were excluded if they underwent neoadjuvant chemotherapy, concurrent radiotherapy, chemotherapy, or conversion therapy before the index day.

### Classification of patient group

The eligible patients were divided into the GST and non-GST groups according to whether the GST stapler was used in the surgeries. All patients who underwent their first laparoscopic gastrectomy using the GST system were assigned to the GST group, and the patients who underwent their first laparoscopic gastrectomy using a non-GST system were assigned to the non-GST group.

### Baseline variables

The demographic variables included gender, age, body mass index (BMI), types of insurance (e.g., urban residence or self-pay), height, weight. The clinical variables included past surgical history, drinking history, comorbidities (e.g., diabetes, hypertension, chronic kidney disease, cardiovascular diseases, other carcinomas/tumors), and pathological stages of GC.

### Outcomes

The primary clinical outcome measures were the stapler-related complications including intraoperative bleeding (defined as the immediate bleeding following the use of stapler), postoperative bleeding (defined as the color of the gastric drainage tube was fresh red), postoperative leakage (defined as anastomotic leakage diagnosed by angiography and X-ray diagnosis), intraoperative anastomosis intervention, intraoperative suture, intraoperative compression, and intraoperative electrocoagulation. The secondary clinical outcome measures included the proportion of postoperative drainage duration longer than eight days. The healthcare utilization outcome measures included operation time in minutes, the total length of stay in days, length of stay after operation in days, cartridge consumption in numbers, and the 30-days readmission rate.

### Statistical analyses

Means and standard deviations were used to report all the normally distributed continuous variables. For variables that were not normally distributed, medians and interquartile ranges were reported. Frequencies and percentages were used to report categorical variables in this study. T-tests, Chi-square tests, and the Mann‐Whitney U‐tests were used to compare the baseline characteristics between the GST and non-GST groups, where appropriate. Multivariate logistic regression was conducted for primary clinical, secondary clinical, and healthcare utilization outcomes to study the effect of variables, controlling for the baseline differences between the two groups. In addition, multivariate logistic regression was also used for the purpose of retaining the maximum study sample size. All the statistical analyses were performed using SAS 9.4 TS Level 1M4.

## Results

Among the 156 screened patients, 121 patients were included in the analysis (Fig. 1), including 59 patients in the GST group and 62 patients in the non-GST group. Mean ages were 57.2 (standard deviation (SD) = 10.97) and 58.5 (SD = 10.44) years old for GST and non-GST group, respectively. The two most common comorbidities were hypertension (GST group: 40.6% vs. non-GST group: 43.8%) and diabetes (GST group: 25%vs. non-GST group: 14.6%). There were no significant group differences in demographic and clinical characteristics (see Table 1), except for the operation method and surgeons who conducted the surgeries (p < 0.0001). No statistically significant group differences in drinking and medical history were observed.

**Table 1:** Baseline social-economic and clinical characteristics (n = 121)

Characteristics	n (%) or mean ± SD		P
	GST (n = 59)	Non-GST (n = 62)	
Age	57.2 ± 11	58.5 ± 10.4	0.29
BMI	23.4 ± 2.8	23.7 ± 3.04	0.53
Male	39 (66.1%)	45 (72.6%)	0.44
Operation method			
TLS	50 (84.7%)	26 (41.9%)	<0.0001
LAS	9 (15.3%)	36 (58.1%)	
Reconstruction			
Billroth1	0	1 (1.6%)	
Billroth2 +Braun	45 (76.3%)	47 (75.8%)	0.62
Roux-en-Y	14 (23.7%)	14 (22.6%)	
Pathological Stage			
Stage IA	28 (47.5%)	17 (27.4%)	
Stage IB	7 (11.9%)	8 (12.9%)	
Stage IIA	5 (8.5%)	4 (6.5%)	
Stage IIB	6 (10.2%)	14 (22.6%)	0.25
Stage IIIA	10 (16.9%)	12 (19.4%)	
Stage IIIB	2 (3.4%)	4 (6.5%)	
Stage IIIC	1 (1.7%)	3 (4.8%)	
Drinking History Yes	25 (42.4%)	30 (48.4%)	0.51
Previous operation (s)			
Yes	21 (35.6%)	24 (38.7%)	0.72
Comorbidities			
Subgroup	n = 23	n = 33	
Diabetes	8 (25%)	7 (14.6%)	
Hypertension	13 (40.6%)	21 (43.8%)	0.45
Anemia	0	1 (2.1%)	
Cancer	1 (3.1%)	0	
Others	10 (31.3%)	19 (39.6%)	
Reimbursement			
Provincial health care	30 (50.8%)	22 (35.5%)	
Urban health care	22 (37.3%)	24 (38.7%)	0.092
Self-pay	7 (11.9%)	16 (25.8%)	
Surgeons			
Surgeon1	29 (49.2%)	55 (88.7%)	
Surgeon2	4 (6.8%)	0	
Surgeon3	11 (18.6%)	3 (4.8%)	<0.0001
Surgeon4	13 (22.0%)	4 (6.5%)	
Surgeon5	2 (3.4%)	0	

Total Laparoscopic Surgery (TLS); Laparoscopic Assisted Surgery (LAS); Body Mass Index (BMI)

Table 2 shows the comparison results of the primary clinical outcome measures between GST and non-GST groups. Compared to the non-GST group, the GST group had a lower proportion of all the perioperative events except for postoperative bleeding and postoperative leak. The proportion of intraoperative anastomosis intervention in GST and non-GST groups were 62.7% and 88.7%, respectively (p = 0.0008). In addition, GST group has significant lower percentage of intraoperative bleeding compared to non-GST group (18.6% vs. 87.1%, p < 0.0001).

**Table 2:** Perioperative outcomes comparison between GST and non-GST patient group (n = 121)

Events	n (%)				P-value
	GST (n = 59)		Non-GST (n = 62)		
IA intervention proportion	37	(62.7%)	55	(88.7%)	0.0008
IB proportion	11	(18.6%)	54	(87.1%)	< 0.0001
IS proportion	25	(42.4%)	40	(64.5%)	0.015
IE proportion	14	(23.7%)	25	(40.3%)	0.051
IP proportion	12	(20.3%)	26	(41.9%)	0.011
PB proportion	1	(1.7%)		0	0.3
Postoperative leak proportion	2	(3.4%)	1	(1.6%)	0.61

Events	Median (Q1, Q3)		P-value
IA intervention times	1 (0; 2)	2 (1; 3)	< 0.0001
Suture times	0 (0; 1)	1 (0; 1)	0.0099
Electrocoagulation times	0 (0; 0)	0 (0; 1)	0.043
Pression times	0 (0; 0)	0 (0; 1)	0.0059

Intraoperative anastomosis (IA); Intraoperative Bleeding (IB); Intraoperative Electrocoagulation (IE); Postoperative Bleeding (PB); Intraoperative Suture (IS); Intraoperative Compression (IC); Intraoperative Pression (IP)

As some of the outcomes are not normally distributed, we used Mann–Whitney-U Test to test the difference between GST and Non-GST group, therefore, the results are presented in Median (Q1, Q3)

**Fig. 2:**
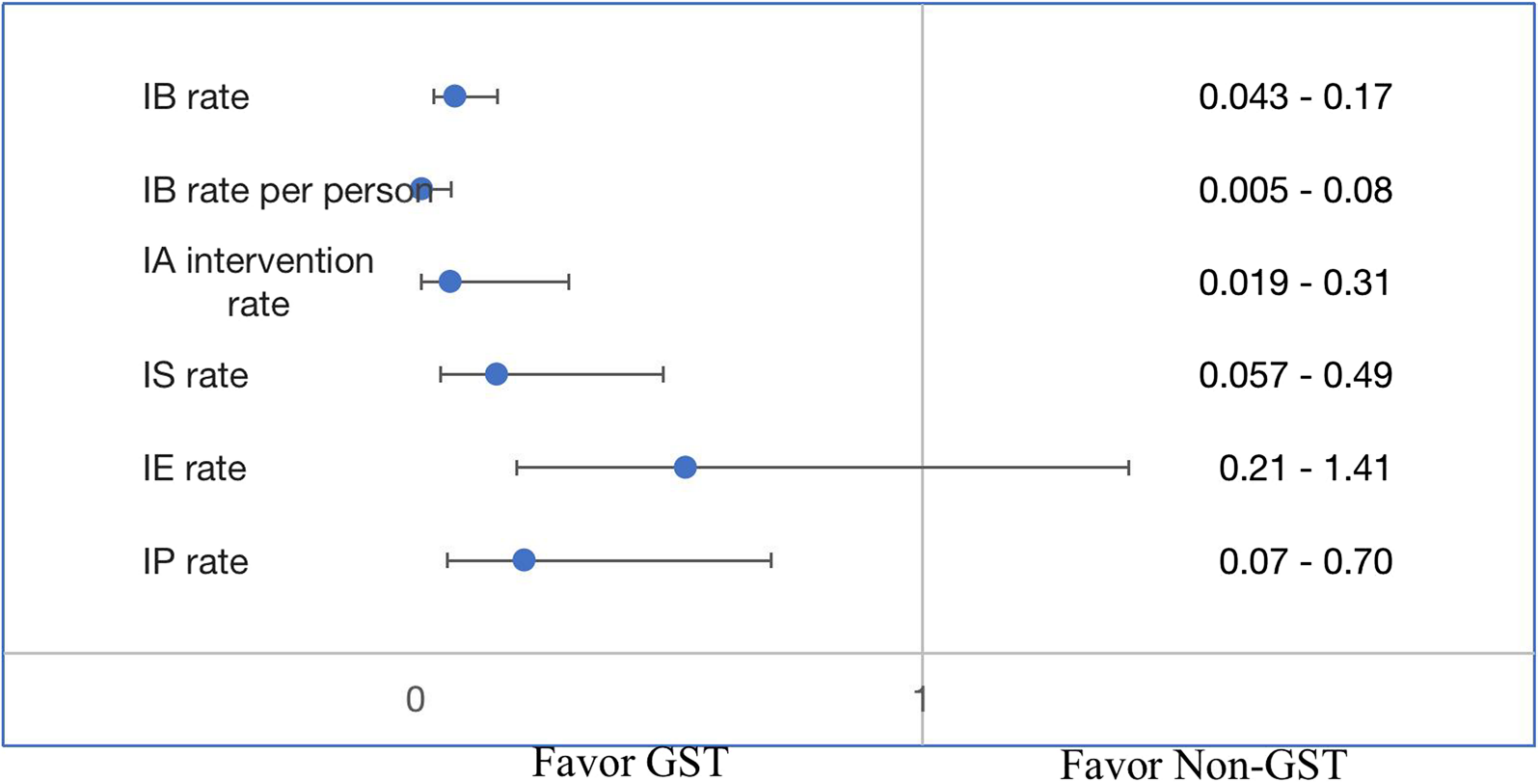
Primary outcomes comparison GST and Non-GST group (in odds ratio and 95% ConfidenceInterval). *Intraoperative anastomosis (IA); Intraoperative Bleeding (IB); Intraoperative Electrocoagulation (IE); Postoperative Bleeding (PB); Intraoperative Suture (IS); Intraoperative Compression (IC); Intraoperative Pression (IP). *Regression model adjusted for age, gender, BMI, comorbidities, and surgeons and operation method

Figure 2 shows the adjusted analysis of the primary clinical outcome measures, compared with the non-GST group, the GST group had a significantly lower risk for intraoperative bleeding (IB) (adjusted odds ratio (aOR): 0.0853 (95% CI 0.0434–0.1675, p < 0.0001), controlling for age, gender, BMI, comorbidities, surgeons and operation method. Age was also a significant risk factor for IB (aOR = 1.0207, 95% CI 1.0005–1.0413, p = 0.0449). The GST group was also associated with lower risks of intraoperative anastomosis intervention (aOR = 0.076, 95% CI 0.019–0.308, p = 0.0003), intraoperative pression (aOR = 0.221, 95% CI 0.07–0.704, p = 0.0107), and intraoperative suture (aOR = 0.167, 95% CI 0.057 − 0.492, p = 0.0012).

Table 3 presents the analyses of secondary clinical outcomes and healthcare utilization outcomes. No statistically significant group differences were found in the proportion of postoperative drainage duration (PDD) ≥ 8 days, operation time, and 30-days readmission rate. The total length of stay was 17.67 ± 4.2 days in the GST group and 19.47 ± 4.4 days in the non-GST group (p = 0.019). As for cartridge consumption, the GST group had one fewer cartridge than the non-GST group when comparing the median (GST: 5 vs. non-GST: 6). In the multivariate logistic regression analysis, however, after adjusting for the baseline differences, the lower cartridge consumption was not statistically significant.

**Table 3:** Medical resource utilization comparison between GST and non-GST

Variables	Mean ± SD, n (%), or Median (q1, q3)		P
	GST	Non-GST	
Operation time (mins)	230.1 ± 47.2 (n = 59)	234.9 ± 54.8 (n = 62)	0.66
Total LOS (d)	17.7 ± 4.2 (n = 55)	19.5 ± 4.4 (n = 60)	0.019
LOS after operation (d)	10.3 ± 2.4 (n = 55)	10.7 ± 2.6 (n = 60)	0.13
Postoperative drainage duration>=8 days	44 (77.2%) (n = 57)	49 (80.3%) (n = 61)	0.6771
(%)			
30-day readmission rate	0 (0%) (n = 57)	2 (3.4%) (n = 58)	0.4957
The number of cartridges used	5 (5; 6)	6 (5; 6)	0.0241

*LOS* Length of stay

## Discussion

To our knowledge, this study was the first real-world study in China to report the effect of the GST system on the perioperative outcomes of gastrectomy procedures. Even though we had a relatively limited sample size, we still observed that the use of the GST system was associated with statistically significantly better intraoperative outcomes, compared to the non-GST group, most of which used manual stapler in surgeries. The risk of intraoperative bleeding was markedly decreased by the GST system, compared to the non-GST group. The GST system and non-GST system did not differ in intraoperative anastomosis intervention, intraoperative suture, and intraoperative compression. There was no statistically significant difference between the two groups in terms of intraoperative electrocoagulation and postoperative leakage. Regarding the economic performance, we found that the use of the GST system was associated with a shorter total length of stay and lower cartridge consumption.

Our study contributes to the knowledge of the real-world effectiveness and economic performance of the GST system. The effect of the GST system on perioperative outcomes in our study is consistent with the previous studies that focused on the effectiveness of the GST system and powered stapler. One study conducted by Logan Rawlins et al. reported that the use of the GST system was associated with a lower risk of hemostasis-related complications such as bleeding and transfusion compared to SigniaTM Stapling System in laparoscopic sleeve gastrectomy surgery [14]. Similarly, a study by Fegelman et al. also reported that the use of the GST system reduced the need for staple line interventions in laparoscopic sleeve gastrectomy [9]. Other researchers also reported that the use of powered staplers was associated with better clinical outcomes compared to the use of manual staplers [9, 10]. While other researchers may focus more on postoperative clinical outcomes [7, 8, 15], our study provided evidence in intraoperative clinical outcomes.

Even though surgical staplers were very helpful to surgeons, perioperative complications were still caused by the technical errors or operation difficulties of manual staplers [11, 12, 16]. Understanding those technical barriers preventing surgeons from having desired clinical outcomes may help us understand why the powered surgical stapler with the GST system could help surgeons perform better in surgeries. The very straightforward technical issues with the manual stapler were the size and the weight of the stapler. Prachi Rojatkar et al. reported that powered staplers only require 3% of the force that is needed by the manual stapler to fire during the surgery, which made it much easier for surgeons to stabilize the stapler device and thus reduced tissue slippage during the stapling process. Despite the superior performance of powered staplers over manual staplers in many aspects, the choice of staplers ultimately rested on the experience and preferences of surgeons. Older surgeons, for instance, might favor manual staplers due to their historical reliance on this option in the absence of powered alternatives, which also explains the mixed selections in the non-GST group. In our study, the GST stapler was easier to hold and operate because of its lighter weight and its double pressurization system. The GST system first performs precompression to the target tissue to squeeze the target tissue into a proper height and then compress the staples to into a B-shape within the tissue, which is thought to be the optimal shape [17]. In addition, the GST stapler can provide a consistent compressive force and gripping force at the same time via its cartridge to the target tissue, which can further reduce tissue slippage. Hence, it was reasonable to observe better clinical outcomes in the GST group in our study. Furthermore, considering the mixed choices of staplers within the non-GST group, it is possible that the impact of GST-powered staplers in our study might be underestimated when compared to manual staplers.

For the economic outcomes of using the powered stapler, many researchers reported that better economic performance was observed [10, 15, 18]. In our study, we observed a notable reduction in the length of hospital stays among patients in the GST group, indicating faster recovery without significant post-operative complications necessitating inpatient admission when compared to individuals in the non-GST group. Moreover, there was no statistically significant difference in the 30-day readmission rates between the two groups. In addition, our investigation revealed a reduced consumption of cartridges in the GST group when compared to the non-GST group. This aligns with our clinical findings, providing additional evidence of the benefits associated with the GST system's implementation. When we looked at the regression results, however, the GST group was not statistically significantly associated with lower consumption of cartridges (p = 0.0501), probably due to the relatively small sample size in our study.

## Limitations

Selection bias is the major limitation. We cannot tell if the true effect of variables of interest was hampered by potential confounding effect due to the non-randomized study design. Because all participants were from the FHHMU and thus the study population was not representative as all participants were from a single clinical site. In addition, the effect of the GST system that we observed in this study can also be influenced by the patients’ preferences for physicians and physicians’ preferences for operation methods. TLS and LAS can directly affect the perioperative outcomes of gastrectomy and more experienced physicians are more favored as they can perform better than novice physicians. Therefore, the effect of the GST system could be either overstated or understated due to patients’ selections and physicians’ selections. A possible way to address this issue is to use propensity score matching to adjust for the differences between the groups, however, this approach was constrained by the relatively small sample size of this study. Hence, the results of this study should be interpreted with caution. Besides, even though we are quite confident that most of our collected information was accurate, information bias may still exist due to the nature of the retrospective study design. Moreover, a causal linkage could not be drawn between the use of the GST system and better clinical outcomes as this study design was an observational retrospective cohort study.

## Conclusion

The use of the GST system for gastrectomy for gastric carcinoma was associated with better clinical outcomes, lower cartridge consumption, and shorter total length of stay compared to the use of a non-GST system in a Chinese real-world setting. Future prospective clinical studies are needed to evidence our findings.

## Data Availability

All authors understand that submission of a manuscript to a BMC journal implies that materials described in the manuscript, including all relevant raw data, will be freely available to any scientist wishing to use them for non-commercial purposes, without breaching participant confidentiality.
